# Association between oxidative balance score and cardiovascular diseases: mediating analysis of methylmalonic acid based on the NHANES database

**DOI:** 10.3389/fnut.2024.1476551

**Published:** 2024-11-11

**Authors:** Xinyu Yang, Zisang Zhang, Fei Ye, Pengfei Liu, Bo Peng, Teng Wang

**Affiliations:** Department of Cardiology, Affiliated Kunshan Hospital of Jiangsu University, Kunshan, Jiangsu, China

**Keywords:** oxidative balance score, cardiovascular diseases, methylmalonic acid, mediating effect, hypertensive patients

## Abstract

**Aim:**

To explore the association between oxidative balance score (OBS) and cardiovascular diseases (CVD) in patients with hypertension, and further clarify the mediating role of methylmalonic acid (MMA) in the relationship between OBS and CVD risk.

**Methods:**

We included 4,137 participants with hypertension from the 2011–2014 National Health and Nutrition Examination Survey cohort. The study endpoint was the incidence of CVD in patients with hypertension. OBS was calculated based on 16 dietary and 4 lifestyle components. Weighted multivariable logistic regression models were adopted to assess the associations between OBS and CVD risk, OBS and MMA levels, and MMA levels and CVD risk. Odds ratios (OR) and 95% confidence interval (CI) were estimated. We used distribution-of-product method to test for mediation effect, with a presence of mediation indicated by 95% CI that does not include 0 for the distribution-of-product method and 95% CI that does not include 1 for the indirect effect.

**Results:**

Totally 812 developed CVD. In weighted multivariable logistic regression models, lower OBS category (OBS < 15.72) was associated with increased odds of CVD (OR = 1.53, 95%CI: 1.17–2.01) and MMA levels (OR = 1.32, 95%CI: 1.06–1.65), respectively, compared with higher OBS category as reference. A positive relationship between higher MMA levels (≥154.90 nmol/L) and CVD risk was observed (OR = 1.34, 95%CI: 1.07–1.68). Importantly, according to the distribution-of-product test, a potential mediating effect of MMA on the relationship between OBS and CVD was found (OR = 1.08, 95%CI: 1.01–1.19), with a 95% CI for distribution-of-product of 0.08 (95% CI: 0.01–0.17). The mediated proportion was 17.8%. Subgroup analysis revealed a mediating effect of MMA in individuals with dyslipidemia, with a mediated proportion of 14.9%.

**Conclusion:**

MMA plays a critical mediating role in the pathway between OBS and CVD risk.

## Introduction

Hypertension is a prevalent chronic disease and is considered as a leading preventable risk factor for cardiovascular diseases (CVD) (1). CVD is known as a primary cause of mortality and disability in humans, a major economic burden globally and a significant contributor to rising healthcare costs (2). The annual cost of CVDs in the European Union is estimated to be €282 billion (3). Preventing or controlling the occurrence and progression of CVDs has become crucial.

There is growing evidence that oxidative stress (OS), which is caused by an imbalance between reactive oxygen species (ROS) production capacity and antioxidant capacity, is a risk factor for CVD (4, 5). The Oxidative balance score (OBS), which is derived from factors including dietary nutrient intake, physical activity, tobacco exposure, alcohol consumption, and body mass index (BMI), serves as a valuable tool for assessing an individual’s OS status (6, 7). Cheng et al. found a negative association between OBS and risk of ischemic heart disease in the general population (8). The study by Wang et al. used multivariate logistic regression analysis to investigate the relationship between OBS and 10-years risk of atherosclerotic cardiovascular disease (ASCVD) in the general population, finding an association between higher OBS and a lower 10-years ASCVD risk (9). However, few studies have assessed the relationship between OBS and CVD risk in patients with hypertension.

Methylmalonic acid (MMA) serves as an intermediate metabolite in the catabolism of four amino acids and odd-chain fatty acids, and there is mounting evidence indicating its pivotal role in mitochondrial dysfunctions and OS (10). This is partially attributed to its ability to disrupt the regulation of the mitochondrial respiratory chain and trigger the release of ROS (10). The development of CVD is primarily attributed to OS and mitochondrial dysfunction (4, 11). A previous study has demonstrated an independent association between elevated serum MMA levels and CVD in the general population (12). However, to date, few studies have assessed the relationship between MMA and CVD development in individuals with hypertension. Recent evidence shows that OBS may affect depression (13) and cognitive function (14) by influencing OS biomarkers, such as blood uric acid and gamma glutaminase transferase. Therefore, we hypothesized that OBS might affect CVD risk in patients with hypertension partly through MMA.

In this study, we aimed to explore the association between OBS and CVD risk in patients with hypertension and to further analyze the potential mediating role of MMA in this relationship, so as to provide valuable insights into the prevention and control of CVD in hypertensive patients.

## Methods

### Study design and participants

Data were obtained from National Health and Nutrition Examination Survey (NHANES) database in this cross-sectional study. NHANES is an ongoing cross-sectional survey using a complex, multistage, and probabilistic sampling design (15). Data collection for the NHANES survey integrates questionnaire surveys, including demographic, socioeconomic, dietary and health-related questions, as well as physical examinations, such as medical, dental, physiological measurements, and laboratory tests. All NHANES participants provided written consent to participate in the survey, and their data collection was approved by the National Center for Health Statistics Research Ethics Review Board.

**Figure 1:**
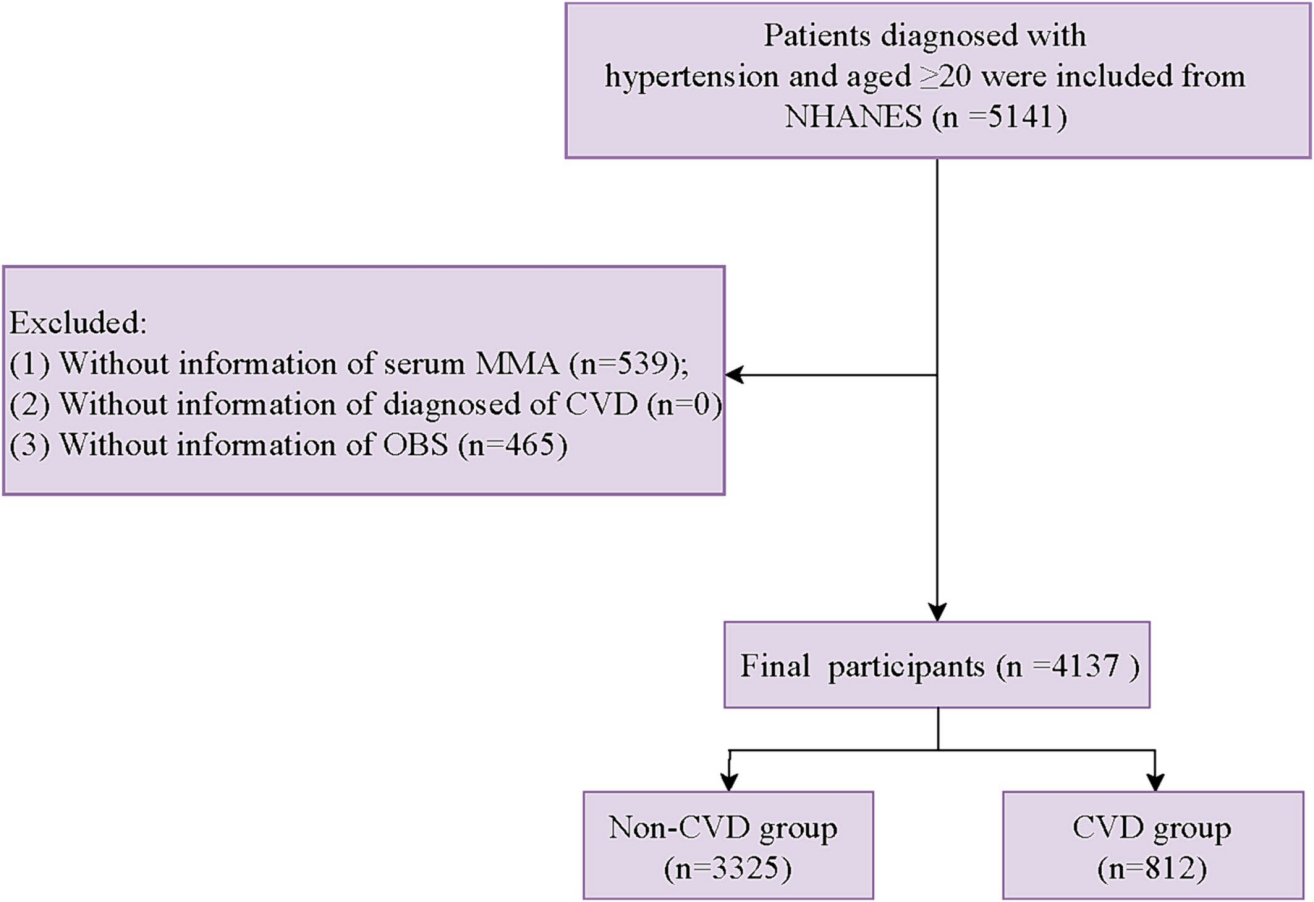
Flow chart of participants’ selection.

Participants diagnosed with hypertension and older than 20 years old from NHANES 2011–2014 (*n* = 5,141) were eligible for the study, while participants without information of serum MMA (*n* = 539), diagnosis of CVD (*n* = 0), and calculating OBS (*n* = 465) were excluded. A total of 4,137 participants were included in the final analysis (Figure 1).

### Outcome variable

The study endpoint was the incidence of CVD in patients with hypertension. CVD was determined by self-reported physician diagnosis: Has a doctor or other health professional ever told you that you had coronary heart disease (CHD)/ angina/angina pectoris/ heart attack/ congestive heart failure (CHF)/ stroke?.” A positive response to these questions suggested that the individual was considered to have CVD (16).

### Independent variable

The OBS was calculated on the basis of 16 dietary and 4 lifestyle components (including 5 pro-oxidants and 15 antioxidants) (17). All factors, except for alcohol, were divided into gender-specific tertiles. Pro-oxidants included total fat, total iron intake, tobacco exposure, alcohol consumption, and BMI (rated 2 points, 1 points, 0 points from the lowest to the highest tertile, respectively). All antioxidants were rated 0, 1, and 2 points from the lowest to the highest tertile. Dietary data in the NHANES was derived from 24 h dietary recall. Lifestyle factors included physical activity, tobacco exposure, alcohol consumption, and BMI. Physical activity was measured by metabolic equivalent (MET) score. Tobacco exposure was assessed by serum cotinine levels. Alcohol consumption was categorized into three groups: nondrinkers (2 points), moderate drinkers (1 point; 0–15 g/day for female or 0–30 g/day for male), and heavy drinkers (0 point; ≥15 g/day for female or ≥ 30 g/day for male). The total OBS score was calculated by summing all factor scores, with higher OBS indicating greater antioxidants exposure. Based on previous literature (18, 19), OBS score was typically stratified into three groups based on tertiles, and the second and third tertile were commonly associated with study outcomes. According to the requirements of subsequent mediation analysis, we used the exposure variable-OBS score as a binary and a continuous variable in this study. Thus, OBS score were divided into two groups based on the tertiles: <15.72 (T1) and ≥ 15.72 (T2 + T3).

### Mediator

Blood samples of participants were collected by venipuncture in mobile examination centers (20). Serum MMA was determined by gas chromatography/mass spectrophotometry. In this study, the association between MMA and CVD risk was found to be nonlinear (*P*-overall < 0.0001 and *P*-nonlinear < 0.0001) using restricted cubic splines (RCS) analysis, with an inflection point observed at 154.90 nmol/L (Supplementary Figure S1). To determine the appropriate cutoff value, we selected the closest value to the inflection point, which corresponded to the median of MMA level. Consequently, we divided MMA levels into two groups based on this median: <154.90 nmol/L and ≥ 154.90 nmol/L.

### Covariates

The following variables were included: age, gender (Male/Female), race (Mexican American, other Hispanic, Non-Hispanic White, Non-Hispanic Black, and others), education level [less than 9th grade/9–11th grade (includes 12th grade with no diploma); high school grade/general equivalent diploma (GED) or equivalent; some college or associate degree, and college graduate or above], poverty income ratio (PIR, <1 and ≥ 1), diabetes (No/Yes), dyslipidemia (No/Yes), white blood cells (WBC, 1000 cells/uL), vitamin B_12_ (pmol/L), and energy (kcal).

### Statistical analysis

Continuous variables were expressed as weighted means (standard errors, SE), and categorical variables were presented as frequencies (weighted percentages). Differences in characteristics were compared using chi-square tests for categorical variables and *t*-tests for continuous variables. Weighted univariable logistic regression analyses were used to screen for potential confounding factors associated with CVD risk (Supplementary Table S1). Weighted multivariable logistic regression models were used to assess the associations between OBS and CVD risk, OBS and MMA, and MMA and CVD risk. Model I ^(a)^ was adjusted for age, gender, and race. Model II ^(a)^ was adjusted for age, gender, race, education level, PIR, diabetes, dyslipidemia, WBC, and energy. Odds ratios (OR) and 95% confidence intervals (CI) was estimated.

Subsequently, weighted multivariate logistic regression was conducted to explore the mediating effect of MMA in the association between OBS and CVD risk. We used distribution-of-product method to examine the mediation effect, with a presence of mediation indicated by a 95% CI that does not include 0 for the distribution-of-product method and a 95% CI that does not include 1 for the indirect effect. Model II ^(b)^ was adjusted for age, gender, race, education level, PIR, diabetes, dyslipidemia, WBC, energy, and MMA, with OR as a direct effect. Additionally, we also explored the interaction of OBS and MMA on CVD risk using weighted multivariate logistic regression. Lastly, we also explored the mediating role of MMA in subgroups of diabetes and dyslipidemia. *p* < 0.05 was considered significant. R version 4.3.2 was used for statistical analysis. Missing variables including education level, WBC, and vitamin B_12_ were interpolated using multiple imputation, and the results of sensitivity analysis are showed in the Supplementary Table S2.

## Results

### Characteristics of study population

The final sample included 4,137 participants with hypertension, with a mean (SE) age of 57.11 (0.34) years. Table 1 shows the characteristics of the participants. 49.22% of the participants were male. The proportion of dyslipidemia was 83.04% (*n* = 3,419). The mean (SE) for vitamin B_12_ was 473.28 (9.23) pmol/L. Of the 4,137 participants, 812 developed CVD. Participants in the CVD group were more likely to be older, have lower educational levels and energy, a higher prevalence of diabetes and dyslipidemia, higher levels of WBCs.

**Table 1:** Characteristics of study population.

Variables	Total (*n* = 4,137)	Non-CVD (*n* = 3,325)	CVD (*n* = 812)	Statistics	*p*
MMA, *n* (%)				χ^2^ = 69.282	<0.001
<154.90 nmol/L	2,132 (49.34)	1843 (52.45)	289 (34.94)		
≥154.90 nmol/L	2,005 (50.66)	1,482 (47.55)	523 (65.06)		
OBS, *n* (%)				χ^2^ = 17.607	<0.001
≥15.72	2,692 (69.97)	2,215 (71.46)	477 (63.07)		
<15.72	1,445 (30.03)	1,110 (28.54)	335 (36.93)		
Age, Mean (S.E)	57.11 (0.34)	55.21 (0.38)	65.89 (0.67)	*t* = −13.33	<0.001
Gender, *n* (%)				χ^2^ = 3.521	0.061
Male	2,050 (49.22)	1,607 (48.28)	443 (53.56)		
Female	2,087 (50.78)	1,718 (51.72)	369 (46.44)		
Race, *n* (%)				χ^2^ = 8.127	0.087
Mexican American	389 (5.76)	325 (6.01)	64 (4.61)		
Other Hispanic	357 (4.44)	299 (4.63)	58 (3.55)		
Non-Hispanic White	1,815 (70.92)	1,385 (70.06)	430 (74.87)		
Non-Hispanic Black	1,136 (12.84)	936 (13.19)	200 (11.25)		
Other Race-Including Multi-Racial	440 (6.04)	380 (6.11)	60 (5.72)		
Education level, *n* (%)				χ^2^ = 22.434	<0.001
Less than 9th Grade/9–11th Grade (Includes 12th grade with no diploma)	1,041 (17.64)	788 (16.48)	253 (23.01)		
High School Grad/GED or Equivalent	989 (23.07)	775 (22.22)	214 (26.98)		
Some College or associate degree/College Graduate or above	2,107 (59.29)	1,762 (61.30)	345 (50.01)		
PIR, *n* (%)				χ^2^ = 8.501	0.014
0–1	837 (13.48)	636 (12.69)	201 (17.11)		
≥1	2,985 (80.56)	2,429 (81.14)	556 (77.90)		
Unknown	315 (5.96)	260 (6.17)	55 (4.99)		
Diabetes, *n* (%)				χ^2^ = 74.435	<0.001
No	2,828 (73.83)	2,400 (77.42)	428 (57.25)		
Yes	1,309 (26.17)	925 (22.58)	384 (42.75)		
Dyslipidemia, *n* (%)				χ^2^ = 44.278	<0.001
No	718 (16.96)	655 (19.17)	63 (6.80)		
Yes	3,419 (83.04)	2,670 (80.83)	749 (93.20)		
WBC (1,000 cells/uL), Mean (S.E)	7.35 (0.07)	7.32 (0.08)	7.53 (0.07)	*t* = −2.10	0.044
Serum vitamin B_12_ (pmol/L), Mean (S.E)	473.28 (9.23)	468.99 (11.04)	493.09 (15.43)	*t* = −1.22	0.233
Energy (kcal), Mean (S.E)	2102.24 (17.90)	2141.26 (19.46)	1922.01 (50.46)	*t* = 4.07	<0.001

CVD, cardiovascular disease; OBS, oxidative balance score; GED, general equivalent diploma; PIR, poverty income ratio; WBC, white blood cells; SE, standard error; χ^2^, chi-square test.

### Relationship between OBS, MMA, and CVD risk

Table 2 displays the association of OBS and MMA with CVD risk by weighted multivariable logistic regression models. After adjusting for age, gender and race, lower OBS category (OBS < 15.72) was found to be associated with an increased incidence of CVD (OR = 1.67, 95%CI: 1.34–2.07) compared with higher OBS category as a reference. After further adjustment for age, gender, race, education level, PIR, diabetes, dyslipidemia, WBC, and energy, the positive relationship between OBS < 15.72 and CVD remained (OR = 1.53, 95%CI: 1.17–2.01). Similar results were observed between MMA ≥ 154.90 nmol/L and CVD in multivariable logistic regression models [Model I ^(a)^: OR = 1.39, 95%CI: 1.15–1.69; Model II ^(a)^: OR = 1.34, 95%CI: 1.07–1.68]. As shown in Table 3, we found the lower level of OBS exhibited a positive relationship with MMA [Model I ^(a)^: OR = 1.32, 95%CI: 1.09–1.58; Model II ^(a)^: OR = 1.32, 95%CI: 1.06–1.65].

**Table 2:** Relationship of OBS and MMA with CVD risk among participants with hypertension.

Variables	Model I ^(a)^		Model II ^(a)^	
	OR (95%CI)	*p*	OR^1^ (95%CI)	*p*
OBS, *n* (%)				
≥15.72	Ref		Ref	
<15.72	1.67 (1.34–2.07)	<0.001	1.53 (1.17–2.01)	0.003
MMA, *n* (%)				
<154.90	Ref		Ref	
≥154.90	1.39 (1.15–1.69)	0.001	1.34 (1.07–1.68)	0.012

CVD, cardiovascular disease; OBS, oxidative balance score; MMA, methylmalonic acid; OR, odds ratios; CI, confidence intervals; Ref, reference. Model I ^(a)^: adjusted for age, gender, and race. Model II ^(a)^: adjusted for age, gender, race, education level, poverty income ratio, diabetes, dyslipidemia, white blood cells, and energy.

**Table 3:** Relationship between OBS and MMA.

Variables	Model I^(a)^		Model II ^(a)^	
	OR (95%CI)	*p*	OR (95%CI)	*p*
OBS, *n* (%)				
≥15.72	Ref		Ref	
<15.72	1.32 (1.09–1.58)	0.005	1.32 (1.06–1.65)	0.013

OBS, oxidative balance score; MMA, methylmalonic acid; OR, odds ratios; CI, confidence intervals; Ref, reference. Model I ^(a)^: adjusted for age, gender, and race. Model II ^(a)^: adjusted for age, gender, race, education level, poverty income ratio, diabetes, dyslipidemia, white blood cells, and energy.

### Mediating effect of MMA

**Figure 2:**
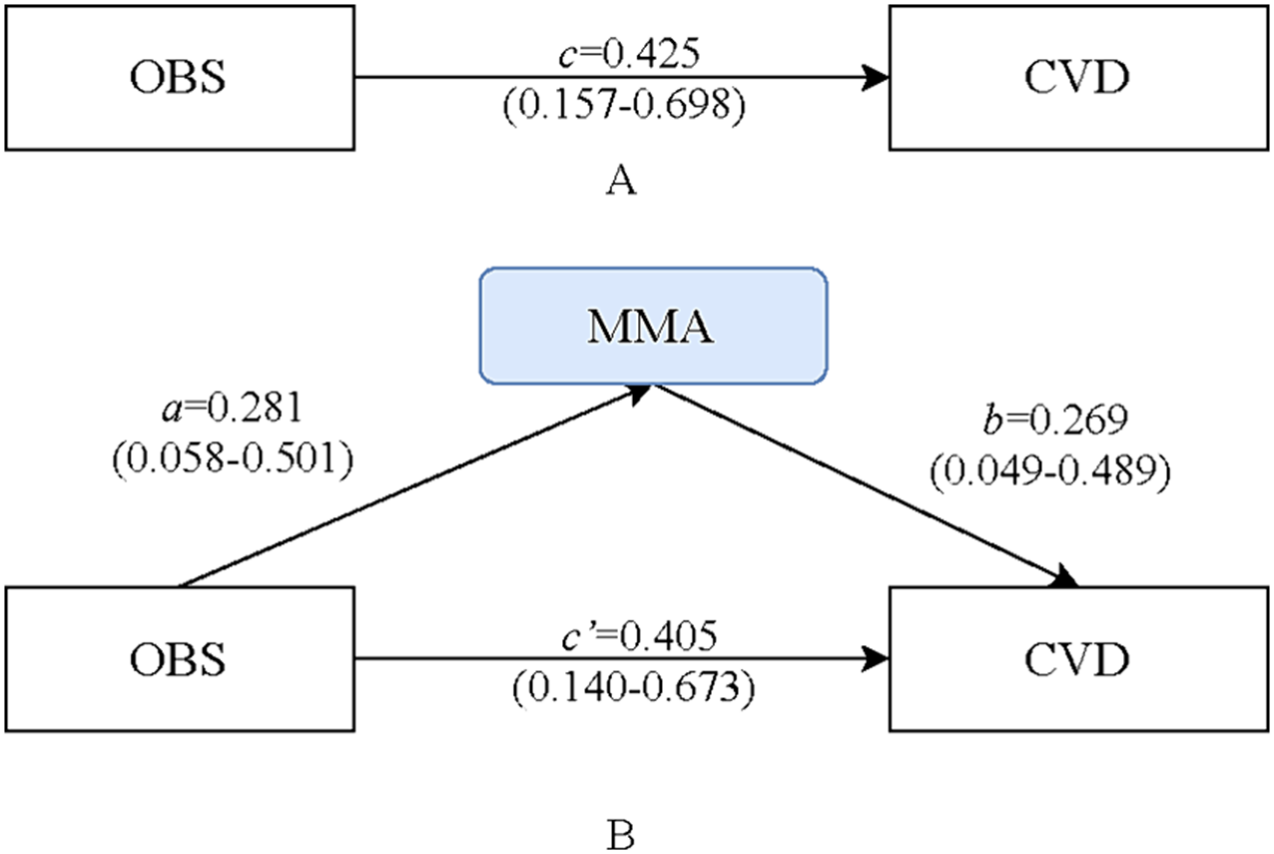
Path diagram of the mediation analysis of methylmalonic acid (MMA) on the relationship between oxidative balance score (OBS) and cardiovascular diseases (CVD). **(A)** the model of total effect on OBS and the risk of CVD, *c* is the estimation coefficient relating the OBS and the risk of CVD; **(B)** the mediation model of MMA on OBS and the risk of CVD, *a* is the estimation coefficient relating the OBS to MMA, and *b* is the estimation coefficient relating the MMA to the risk of CVD adjusted for the OBS, and *c’* is the estimation coefficient relating the OBS to the risk of CVD adjusted for MMA.

The mediating effect of MMA between OBS and CVD are presented in Table 4. The OR for the total effect of OBS and CVD was 1.53 (95%CI: 1.17–2.01). After further adjustment for MMA based on Model II ^(a)^, the association between OBS and CVD was significant [direct effect; Model II ^(b)^ OR = 1.50, 95%CI: 1.15–1.96]. Based on distribution-of-product test, a potential mediating effect of MMA on the relationship between OBS and CVD was observed (OR = 1.08, 95%CI: 1.01–1.19), with a 95% CI for distribution-of-product of 0.08 (95% CI: 0.01–0.17). The mediated proportion was 17.8% (Figure 2). As shown in Supplementary Table S3, we also found that even with OBS as a continuous variable, MMA still mediated the association between OBS and CVD risk, with a mediated proportion of 19.33%. Subgroup analyses revealed that the mediating effect of MMA was observed only in individuals with dyslipidemia, with a mediated proportion of 14.9% (Table 4). Additionally, we used weighted multivariate logistic regression analysis to assess the interaction effect of OBS and MMA on CVD risk. After adjusting for all covariates, we found no significant interaction effect of OBS and MMA on CVD risk, suggesting a reliable mediation (Table 5).

**Table 4:** Mediating effect of MMA on the association between OBS and CVD.

Populations	Model II ^(a)^ (total effect)	Model II ^(b)^ (direct effect)	Distribution-of-product	Indirect effect	Mediated proportion (%)
	OR (95% CI)	OR (95% CI)	β (95% CI)	OR (95% CI)	
Total	1.53 (1.17–2.01)	1.50 (1.15–1.96)	0.08 (0.01, 0.17)	1.08 (1.01–1.19)	17.8
Non-diabetes	1.33 (0.91–1.94)	1.31 (0.90–1.91)	0.05 (−0.04, 0.17)	1.05 (0.96–1.19)	–
Diabetes	1.88 (1.29–2.75)	1.87 (1.28–2.75)	0.06 (−0.11, 0.26)	1.06 (0.90–1.30)	–
Non-dyslipidemia	1.04 (0.41–2.64)	1.01 (0.40–2.58)	0.077 (−0.269, 0.528)	1.080 (0.764–1.696)	–
Dyslipidemia	1.61 (1.22–2.13)	1.58 (1.20–2.08)	0.071 (0.003, 0.174)	1.074 (1.003–1.190)	14.9

CVD, cardiovascular disease; OBS, oxidative balance score; MMA, methylmalonic acid; OR, odds ratios; CI, confidence intervals; Ref, reference. Model II ^(a)^: adjusted for age, gender, race, education level, poverty income ratio, diabetes, dyslipidemia, white blood cells, and energy. Model II ^(b)^: adjusted for age, gender, race, education level, poverty income ratio, diabetes, dyslipidemia, white blood cells, energy, and MMA.

**Table 5:** The interaction effect of OBS and MMA on CVD.

Populations	Variables	Model II ^(a)^	
		OR (95% CI)	*p*
Total	MMA	1.36 (1.03–1.81)	0.031
	OBS	1.63 (0.99–2.66)	0.052
	MMA*OBS	0.88 (0.53–1.47)	0.624
Non-diabetes	MMA	1.24 (0.94–1.65)	0.129
	OBS	1.54 (0.79–3.00)	0.200
	MMA*OBS	0.78 (0.39–1.54)	0.457
Diabetes	MMA	1.56 (0.94–2.58)	0.082
	OBS	1.85 (0.92–3.73)	0.082
	MMA*OBS	1.02 (0.49–2.11)	0.957
Non-dyslipidemia	MMA	1.13 (0.54–2.38)	0.742
	OBS	0.98 (0.31–3.11)	0.973
	MMA*OBS	1.06 (0.29–3.96)	0.924
Dyslipidemia	MMA	1.38 (1.01–1.90)	0.045
	OBS	1.75 (1.01–3.02)	0.045
	MMA*OBS	0.85 (0.48–1.52)	0.574

CVD, cardiovascular disease; OBS, oxidative balance score; MMA, methylmalonic acid; OR, odds ratios; CI, confidence intervals; Ref, reference. Model II ^(a)^: adjusted for age, gender, race, education level, poverty income ratio, diabetes, dyslipidemia, white blood cells, and energy (For total populations). Model II ^(a)^: adjusted for age, gender, race, education level, poverty income ratio, dyslipidemia, white blood cells, and energy (For diabetes and non-diabetes subgroups). Model II ^(a)^: adjusted for age, gender, race, education level, poverty income ratio, diabetes, white blood cells, and energy (For dyslipidemia and non-dyslipidemia subgroups).

## Discussion

In the present study with a nationally representative sample with hypertension, we assessed the relationship between OBS, MMA, and CVD risk, and the mediating role played by MMA. The findings showed that lower OBS was associated with increased risk of CVD and MMA levels, respectively. Additionally, a positive relationship between higher MMA levels and CVD risk was observed. Of particular significance, a novel finding suggests that MMA may significantly mediate the association between OBS and CVD risk. The mediating role of MMA varied by diabetes and dyslipidemia, with subgroup analyses showing partial mediation by MMA (14.9%) in individuals with dyslipidemia and no mediation in other subgroups. These findings may provide potential theoretical references for understanding the effect of OBS on CVD risk in the patients with hypertension through MMA.

To our knowledge, several epidemiological studies have explored the association between OBS and CVD risk. A low OBS score may increase the risk of CVD (21, 22). OBS is a measure of body’s oxidant-antioxidant balance. Disruption of the pro-oxidant-antioxidant balance could result in OS and inflammation (23). It is widely recognized that OS and inflammation are closely related to the onset and progression of CVD events (24). Furthermore, a low OBS score indicates an advantage of pro-oxidant over antioxidant exposure, resulting in elevated levels of OS. OS represents a cascade of adaptive responses triggered by an imbalance between ROS and the antioxidant system (25). ROS readily reacts with small molecules like low-density lipoprotein (LDL), to form oxidized low-density lipoprotein (ox-LDL) (26). Driven by chemokines (e.g., stroma-derived factors and macrophage chemokines), ox-LDL circulates directly to the heart and blood vessels. Under the influence of adhesion factors like plasminogen activator inhibitors, these ox-LDL particles adhere to vascular walls, promote arteriosclerosis and stenosis, and ultimately lead to the development of CVD (9, 27). However, to the best of our knowledge, the majority of current studies have primarily focused on investigating the association between OBS and CVD risk in the general population. However, there is limited research on whether OBS also exerts an impact on CVD risk in individuals with hypertension. Our study involved 4,137 participants with hypertension, and revealed a positive association between lower OBS and CVD risk in participants with hypertension. This finding also means that the interventions of antioxidant-rich diets and lifestyles may be important in reducing the risk of CVD in hypertensive populations.

A positive relationship between lower OBS and MMA levels was also found in this study. To the best of our knowledge, few research has indicated an association between OBS and MMA levels. Only one cross-sectional study reported lower mean MMA values in participants with higher dietary OBS (28). MMA is an intermediate metabolite in the catabolism of four amino acids and odd-chain fatty acids, and is widely recognized as a diagnostic biomarker of vitamin B_12_ deficiency (29). MMA accumulation has the potential to induce oxidative stress, impair mitochondrial function, disrupt cellular energy metabolism, and trigger cell death (30). Lower OBS score means greater oxidant exposure, promoting the accumulation of MMA. As a biomarker of oxidative stress, MMA has been shown to be associated with the risk of CVD (12, 31). Similar results were found in our study, implying that elevated MMA levels in participants with hypertension are positively correlated with CVD risk. In addition to OS, mitochondrial function is crucial for the growth and functionality of endothelial cells in blood vessels (10). Mitochondrial dysfunction adversely affects energy production and cellular physiology, leading to apoptosis, oxidation, and calcium-mediated damage in cardiomyocytes (12). Thus, mitochondrial dysfunction and OS may be potential mechanisms for the association between MMA and CVD risk (31).

It is worth mentioning that we conducted a mediation analysis, and found that MMA significantly mediated the association between OBS and CVD risk in participants with hypertension, with a mediated proportion of 17.8%. This finding also serves as a reminder of the significance of moderately reducing MMA levels to mitigate the onset of CVD, especially in people with hypertension whose diets and lifestyles are rich in pro-oxidants. In addition, we also observed dyslipidemia specificity in the role of MMA in mediating the association between OBS and CVD risk. In hypertensive populations with dyslipidemia, MMA may partially mediate the association between OBS and CVD risk, with a mediated proportion of 14.9%. This may be due to mitochondrial redox dysfunction and mitochondrial energy metabolism abnormalities in a population with dyslipidemia (32). Moreover, our investigation also revealed that over 80% of the study population exhibited dyslipidemia. The results may offer new insights into the development of CVD in complications-specific populations.

Our research had several advantages. Firstly, this was the first study to examine the associations between OBS, MMA, and CVD risk, and the mediating role that MMA plays in hypertensive patients. Secondly, we utilized the nationally renowned NHANES database, which is widely recognized for its extensive sample size and national representation. Thirdly, the association was found to be robust even after adjusting for various important confounders. The present study has some limitations that need to be mentioned. Firstly, this cross-sectional study failed to establish a causal relationship between OBS, MMA, and CVD risk. Further cohort studies are required to confirm it. Secondly, the OBS measurement relied on 24 h dietary recall and the CVD assessment relied on self-reported data, which may introduce a potential recall bias. Additionally, despite our efforts to adjust for several confounders, there may be additional confounders in our analyses due to the limitations of the NHANES database, such as some inflammatory markers (malondialdehyde and nitric oxide) and changes in treatment modalities. Finally, the generalizability of our results may be limited as this study only included participants from the United States.

## Conclusion

In summary, our study revealed significant associations between lower OBS and an elevated risk of CVD, as well as increased levels of MMA in participants with hypertension. Higher MMA was found to be associated with an increased possibility of CVD. Of particular significance, MMA plays a critical mediating role in the pathway between OBS and CVD risk.

## Data Availability

Publicly available datasets were analyzed in this study. This data can be found here: the NHANES database, https://wwwn.cdc.gov/nchs/nhanes/.
