# Supplementary material for: Association between oxidative balance score and cardiovascular diseases: mediating analysis of methylmalonic acid based on the NHANES database
Source: Front Nutr. 2024 Nov 11;11:1476551. doi: 10.3389/fnut.2024.1476551 (PMC11587900; doi:10.3389/fnut.2024.1476551)
Supplement: Supplementary file 2 [file Table_2.DOCX]

Supplementary Table 1 The screening of the potential confounding factors related to CVD

| **Variables** | **OR (95%CI)** | ***P*** |
| --- | --- | --- |
| Age | 1.06 (1.05-1.07) | <0.001 |
| Gender |  |  |
| Male | Ref |  |
| Female | 0.81 (0.64-1.02) | 0.069 |
| Race |  |  |
| Mexican American | Ref |  |
| Other Hispanic | 1.00 (0.68-1.46) | 0.998 |
| Non-Hispanic White | 1.39 (0.98-1.98) | 0.065 |
| Non-Hispanic Black | 1.11 (0.77-1.59) | 0.559 |
| Other Race - Including Multi-Racial | 1.22 (0.69-2.16) | 0.490 |
| Education level |  |  |
| Less Than 9th Grade/9-11th Grade (Includes 12th grade with no diploma) | Ref |  |
| High School Grad/GED or Equivalent | 0.87 (0.65-1.16) | 0.324 |
| Some College or AA degree/ College Graduate or above | 0.58 (0.43-0.79) | <0.001 |
| PIR |  |  |
| 0-1 | Ref |  |
| ≥1 | 0.71 (0.52-0.97) | 0.030 |
| Unknown | 0.60 (0.42-0.86) | 0.007 |
| Diabetes |  |  |
| No | Ref |  |
| Yes | 2.56 (2.03-3.24) | <0.001 |
| Dyslipidemia |  |  |
| No | Ref |  |
| Yes | 3.25 (2.24-4.72) | <0.001 |
| WBC (1000 cells/uL) | 1.04 (1.01-1.08) | 0.036 |
| Serum vitamin B_12_ (pmol/L) | 1.00 (1.00-1.00) | 0.261 |
| Energy (kcal) | 0.99 (0.99-0.99) | <0.001 |

Abbreviations: CVD, cardiovascular disease; GED, general equivalent diploma; PIR, poverty income ratio; WBC, white blood cells.

Supplementary Table 2 Sensitivity analysis

| **Variables** | **Before multiple imputation (n=4137)** | **After multiple imputation (n=4137)** | **Statistics** | ***P*** |
| --- | --- | --- | --- | --- |
| Education, n (%) |  |  | χ^2^=1.932 | 0.381 |
| Less Than 9th Grade/9-11th Grade (Includes 12th grade with no diploma) | 1041 (17.64) | 1041 (17.64) |  |  |
| High School Grad/GED or Equivalent | 989 (23.07) | 989 (23.07) |  |  |
| Some College or AA degree/ College Graduate or above | 2106 (59.29) | 2107 (59.29) |  |  |
| WBC (1000 cells/uL), Mean (S.E) | 7.36 (0.07) | 7.35 (0.07) | t=1.41 | 0.168 |
| Serum vitamin B_12_ (pmol/L), Mean (S.E) | 473.40 (9.23) | 473.28 (9.23) | t=1.78 | 0.084 |

Abbreviations: GED, general equivalent diploma; WBC, white blood cells; SE, standard error; χ^2^, chi‐square test.

Supplementary Table 3 Mediating effect of MMA on the association between OBS (as continuous variable) and CVD

| **Populations** | **Model II ^(a)^**  **(total effect)** | **Model II ^(b)^**  **(direct effect)** | **Distribution-of-product** | **Indirect effect** | **Mediated proportion (%)** |
| --- | --- | --- | --- | --- | --- |
|  | **OR (95% CI)** | **OR (95% CI)** | **β (95% CI)** | **OR (95% CI)** |  |
| Total | 0.97  (0.96-0.99) | 0.98  (0.96-0.99) | -0.0058  （-0.0133, -0.0006） | 0.9942  (0.9868-0.9994) | 19.33 |

Abbreviations: CVD, cardiovascular disease; OBS, oxidative balance score; MMA, methylmalonic acid; OR, odds ratios; CI, confidence intervals; Ref, reference.

Model II ^(a)^: adjusted for age, gender, race, education level, poverty income ratio, diabetes, dyslipidemia, white blood cells, and energy.

Model II ^(b)^: adjusted for age, gender, race, education level, poverty income ratio, diabetes, dyslipidemia, white blood cells, energy, and MMA.
