# Supplementary figures and images for: Association between oxidative balance score and cardiovascular diseases: mediating analysis of methylmalonic acid based on the NHANES database
Source: Front Nutr. 2024 Nov 11;11:1476551. doi: 10.3389/fnut.2024.1476551 (PMC11587900; doi:10.3389/fnut.2024.1476551)

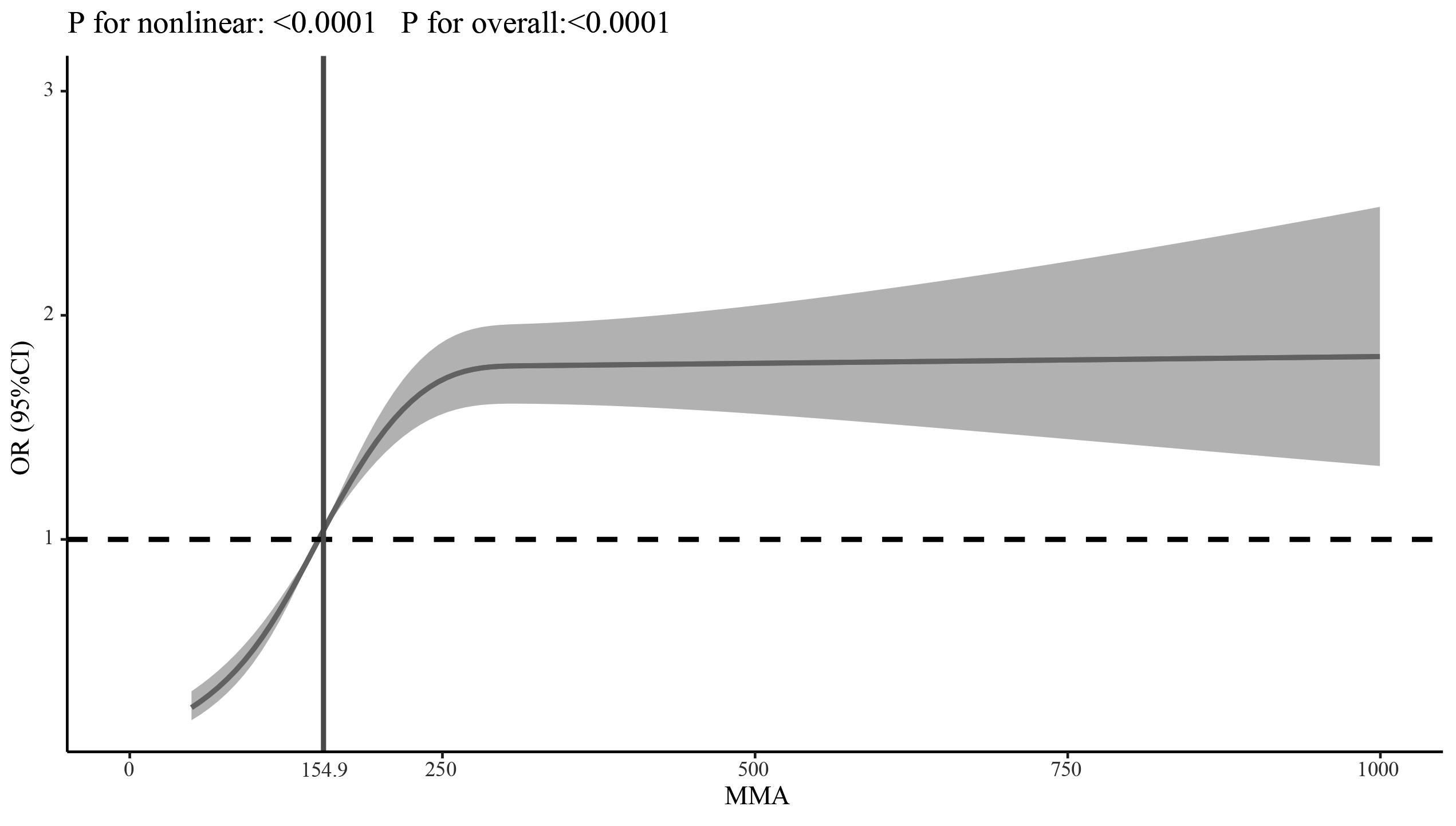

Supplement: Supplementary file 3 [file Image_1.TIF]
