# Supplementary material for: Association between oxidative balance score and cardiovascular diseases: mediating analysis of methylmalonic acid based on the NHANES database
Source: Front Nutr. 2024 Nov 11;11:1476551. doi: 10.3389/fnut.2024.1476551 (PMC11587900; doi:10.3389/fnut.2024.1476551)
Supplement: Supplementary file 1 [file Table_1.DOC]

**Response Letter**

Dear Editor-in-Chief,

Thank you very much for your letter and reviewer’s constructive comments on our manuscript entitled “Association between oxidative balance score and cardiovascular diseases: mediating analysis of methylmalonic acid based on the NHANES database”. We would like to express our sincere thanks to the editor and reviewers about the constructive and positive comments. In this revised version, changes to our manuscript were highlighted by red colored text, and point by point responses to the editor and reviewers are listed below this letter.

We hope that the revised version of the manuscript is now acceptable for publication in your journal.

I look forward to hearing from you soon.

Yours sincerely,

Teng Wang

**Reviewer 1**

The authors explore the association between oxidative balance score (OBS) and cardiovascular diseases (CVD) among patients with hypertension, and further clarify the mediating role of methylmalonic acid (MMA) in the relationship between OBS and CVD risk. Although the study was well-designed, there are still some shortcomings, and the following points are suggested for the authors' consideration.

1.The MMA is categorized according to the median. Is there a theoretical or experimental basis to support this grouping method? This information may have an impact on the interpretation and application of the results, so a description or citation of the literature should be given.
Reply: Thanks very much for your suggestion. In this study, the association between MMA and CVD risk was found to be nonlinear using restricted cubic splines (RCS) analysis (P-overall <0.0001 and P-nonlinear <0.0001), with an inflection point observed at 154.90 nmol/L (Supplementary Figure 1). To determine the appropriate cutoff value, we selected the closest value to the inflection point, which corresponded to the median of MMA level. Consequently, we divided MMA levels into two groups based on this median: <154.90 nmol/L and ≥154.90 nmol/L.

2.When discussing lifestyles, only diet is discussed further, please give reasons for this or add to the discussion as appropriate

Reply: We really appreciate your advice. According to all reviewers’ constructive comments, we have modified the introduction, and please see the revised manuscript.

3. “OBS were divided into two groups based on the tertiles: <15.72 (T1) and ≥15.72 (T2+T3).” Does T1 and T2+T3 mean anything? Not explained in the text. Please revise it.

Reply: We sincerely appreciate your valuable comments. Based on previous literature, OBS score was typically stratified into three groups based on tertiles, and there were commonly associations of the second and third tertiles with the outcome [1-2]. According to the requirements of subsequent mediation analysis, we treated the exposure variable-OBS score as both binary and continuous variables in this study. Thus, OBS score were divided into two groups based on the tertiles: <15.72 (T1) and ≥ 15.72 (T2+T3).

[1] Kwon YJ, Park HM, Lee JH. Inverse Association between Oxidative Balance Score and Incident Type 2 Diabetes Mellitus. Nutrients. 2023;15(11):2497.

[2] Son DH, Lee HS, Seol SY, Lee YJ, Lee JH. Association between the Oxidative Balance Score and Incident Chronic Kidney Disease in Adults. Antioxidants (Basel). 2023;12(2):335.

4.The article mentions that MMA has a mediating role in the dyslipidemia population, but not in other subgroups. It is suggested that the reasons for this phenomenon, as well as its underlying biological mechanisms, should be further explained.
Reply: Thanks very much for your suggestion. According to your advice, we have added the content in the discussion section: For the hypertensive population along with dyslipidemia, MMA may partially mediate the association between OBS and CVD risk with a mediated proportion of 14.9%. This reason may be due to the mitochondrial redox dysfunction and mitochondrial energy metabolism abnormalities in population with dyslipidemia [1]. Moreover, our investigation also revealed that over 80% of the study population exhibited dyslipidemia.

[1] Oliveira HCF, Vercesi AE. Mitochondrial bioenergetics and redox dysfunctions in hypercholesterolemia and atherosclerosis. Mol Aspects Med. 2020; 71:100840.

5.Refinement of the analysis of limitations: It is recommended that the limitations of the study be analysed more comprehensively, e.g. whether there are other potential confounders.

Reply: Thanks very much for your suggestion. According to your advice, we have added the limitations in the discussion section: Additionally, despite our efforts to adjust for several confounders, the possibility of additional con-founding in our analyses due to the limitations of the NHANES database, such as some inflammatory markers (malondialdehyde and nitric oxide) and variations in treatment modalities.

a. Is the quality of the figures and tables satisfactory?
- Yes
Reply: Thanks very much for your review.

b. Does the reference list cover the relevant literature adequately and in an unbiased manner?
- Yes
Reply: Thanks very much for your review.

c. Are the statistical methods valid and correctly applied? (e.g. sample size, choice of test)
- Yes
Reply: Thanks very much for your comments.

d. Is a statistician required to evaluate this study?
- No
Reply: Thanks very much for your review.

e. Are the methods sufficiently documented to allow replication studies?
-Yes

Reply: Thanks very much for your review.

**Reviewer 3**

The present manuscript named “Association between oxidative balance score and cardiovascular diseases: mediating analysis of methylmalonic acid based on the NHANES database” explores the association between oxidative balance score and cardiovascular diseases among patients with hypertension, and further clarifies the mediating role of methylmalonic acid in this association. Therefore, this study provides an interesting update on the development of cardiovascular, however, the manuscript contains several points that the authors need to review:
Introduction
1. In Lines 50-52, the authors stated the global health and economic burden of CVD, which is essential. However, I recommend being more specific by adding the societal cost, if there is, for more precision.

Reply: We sincerely appreciate your valuable comments. We have modified the content in the Introduction section: CVD is known as the primary cause of mortality and disability in humans, representing the leading global economic burden and a significant contributor to elevated healthcare costs (2). According to estimates, the European Union incurs an annual cost of €282 billion due to CVD [1].

[1] Luengo-Fernandez R, Walli-Attaei M, Gray A, Torbica A, Maggioni AP, Huculeci R, Bairami F, Aboyans V, Timmis AD, Vardas P, Leal J. Economic burden of cardiovascular diseases in the European Union: a population-based cost study. Eur Heart J. 2023;44(45):4752-4767.

2. Lines 59-65; the literature review there, about the association between OBS and CVD is not sufficient. Only two studies were mentioned, and both reported negative associations, therefore your statement “The relationship between OBS and CVD risk among patients with hypertension remains unclear”, is not consistent. Please revise this part.

Reply: We sincerely appreciate your valuable comments. However, to the best of our knowledge, the majority of current studies have primarily focused on investigating the association between OBS and CVD risk within the general population. However, limited research has been conducted to explore whether OBS also exerts an impact on CVD risk among individuals with hypertension. We have modified the content in the Introduction section: Cheng et al. found the negative association between OBS and risk of ischemic heart disease among general population [1]. In the study conducted by Wang et al., multivariate logistic regression analysis was employed to investigate the relationship between the OBS and the 10-years risk of atherosclerotic cardiovascular disease (ASCVD) among general population, revealing an association between higher OBS and a reduced 10-years ASCVD risk [2]. However, few studies have assessed the relationship between OBS and CVD risk among patients with hypertension.

[1] Cheng S, Han Y, Jiang L, Lan Z, Liao H, Guo J. Associations of Oxidative Balance Score and Visceral Adiposity Index with Risk of Ischaemic Heart Disease: A Cross-Sectional Study of Nhanes, 2005-2018. BMJ Open (2023) 13(7): e072334.

[2] Wang R, Gao K, Wang L, Gong X, Wu Y, Zheng L, et al. A Cross-Sectional Study Exploring the Relationship between Oxidative Balance Score and 10-Year Atherosclerotic Cardiovascular Disease Risk Based on the National Health and Nutrition Examination Survey (2011-2020). Diab Vasc Dis Res (2024) 21(2):14791641241244658.

3. The association between oxidative stress and CVDs, as well as the association between MMA levels and CVDs, have been extensively documented in the scientific literature. Hence, the basis of your study is supported by only a few previous works, which may impact considerably the objective of the manuscript. I suggest you highlight the need of the study by providing solid support such as previously published work instead of citing only a few when many articles have demonstrated relevant results.

Reply: Thanks very much for your suggestion. In the introduction section, we have provided a preliminary introduction to the relationship between MMA, OBS, and CVD based on previous studies. However, most of the existing studies have primarily focused on the general population, with insufficient attention paid to the CVD risk in hypertensive patients. More importantly, our study aimed to assess whether MMA plays a mediating role in the relationship between OBS and CVD. In the discussion section, we have added more content about the mechanisms, please refer to the revised manuscript.

4. MMA was only briefly mentioned by the authors. Yet, its role in the present study is really significant. More information about MMA is needed, such as its role in the metabolism as it can be helpful to understand its attributed mediating impacts.

Reply: We sincerely appreciate your valuable comments. We have modified the content in the Introduction section: Methylmalonic acid (MMA) serves as an intermediate metabolite in the catabolism of four amino acids and odd-chain fatty acids, with mounting evidence indicating its pivotal role in mitochondrial dysfunctions and OS [1]. This is partially attributed to its ability to disrupt the regulation of the mitochondrial respiratory chain and trigger the release of ROS [1]. The development of CVD is primarily attributed to OS and mitochondrial dysfunction [2-3]. Previous a study has demonstrated an independent association between elevated serum MMA levels and the presence of CVD among general population [4]. However, few studies to date have assessed the relationship between MMA and CVD development in individuals with hypertension.

[1] Lesnefsky EJ, Moghaddas S, Tandler B, Kerner J, Hoppel CL. Mitochondrial dysfunction in cardiac disease: ischemia--reperfusion, aging, and heart failure. J Mol Cell Cardiol. 2001 Jun;33(6):1065-89.

[2] Yan Q, Liu S, Sun Y, Chen C, Yang S, Lin M, et al. Targeting Oxidative Stress as a Preventive and Therapeutic Approach for Cardiovascular Disease. J Transl Med (2023) 21(1):519. Epub 2023/08/03.

[3] Xia D, Liu Y, Wu P, Wei D. Current Advances of Mitochondrial Dysfunction and Cardiovascular Disease and Promising Therapeutic Strategies. Am J Pathol (2023) 193(10):1485-500. Epub 2023/07/23.

[4] Wang X, Li W, Xiang M. Increased serum methylmalonic acid levels were associated with the presence of cardiovascular diseases. Front Cardiovasc Med. 2022 Oct 10; 9:966543.

5. Please provide in line 71, the possible mechanism of MMA on CVD. More details about the possible mechanism of MMA on CVD need to be provided for additional support.

Reply: We sincerely appreciate your valuable comments. We have modified the content in the Introduction section: Methylmalonic acid (MMA) serves as an intermediate metabolite in the catabolism of four amino acids and odd-chain fatty acids, with mounting evidence indicating its pivotal role in mitochondrial dysfunctions and OS [1]. This is partially attributed to its ability to disrupt the regulation of the mitochondrial respiratory chain and trigger the release of ROS [1]. The development of CVD is primarily attributed to OS and mitochondrial dysfunction [2-3]. Previous a study has demonstrated an independent association between elevated serum MMA levels and the presence of CVD among general population [4]. However, few studies to date have assessed the relationship between MMA and CVD development in individuals with hypertension.

More mechanisms are described in the discussion section. Please refer to the revised manuscript.

[1] Lesnefsky EJ, Moghaddas S, Tandler B, Kerner J, Hoppel CL. Mitochondrial dysfunction in cardiac disease: ischemia--reperfusion, aging, and heart failure. J Mol Cell Cardiol. 2001 Jun;33(6):1065-89.

[2] Yan Q, Liu S, Sun Y, Chen C, Yang S, Lin M, et al. Targeting Oxidative Stress as a Preventive and Therapeutic Approach for Cardiovascular Disease. J Transl Med (2023) 21(1):519. Epub 2023/08/03.

[3] Xia D, Liu Y, Wu P, Wei D. Current Advances of Mitochondrial Dysfunction and Cardiovascular Disease and Promising Therapeutic Strategies. Am J Pathol (2023) 193(10):1485-500. Epub 2023/07/23.

[4] Wang X, Li W, Xiang M. Increased serum methylmalonic acid levels were associated with the presence of cardiovascular diseases. Front Cardiovasc Med. 2022 Oct 10; 9:966543.

6. Given the role of HTA in CVD, investigating the potential mediating effect of MMA on the association between OBS and CVD risk among patients with HTA may seem biased. Instead of MMA levels, isn’t it HTA impacting the hypothesized association? Hence, this part needs more highlights.

Reply: We sincerely appreciate your valuable comments. In this study, we aimed to explore the association between OBS and CVD risk among patients with hypertension, and further analyze the potential mediating effect of MMA on this association. The study cohort consisted of individuals diagnosed with hypertension. The mediating effect of MMA on the association between OBS and CVD risk may not be influenced by hypertension. Similar studies have also been reported [1-2].

[1] Zhang X, Wei R, Wang X, Zhang W, Li M, Ni T, Weng W, Li Q. The neutrophil-to-lymphocyte ratio is associated with all-cause and cardiovascular mortality among individuals with hypertension. Cardiovasc Diabetol. 2024 Apr 2;23(1):117.

[2] Zhou W, Sun L, Zeng L, Wan L. Mediation of the association between sleep disorders and cardiovascular disease by depressive symptoms: An analysis of the National health and Nutrition Examination Survey (NHANES) 2017-2020. Prev Med Rep. 2023 Mar 21;33:102183.

Methods
7. Given the inverse relationship between MMA and vitamin B12, as high MMA level is already a sign of a vitamin B12 deficiency, why select vitamin B12 as a cofounder?

Reply: We sincerely appreciate your valuable comments. In this study, weighted univariable logistic regression analysis was used to screen the potential confounding factors that associated with CVD risk (Supplementary Table 1), and *P*<0.05 was considered significant. As shown in Supplementary Table 1, the impact of vitamin B_12_ on CVD did not reach statistical significance; therefore, it was not considered as a confounding factor in this study.

Results
8. In lines 174-175, the authors stated that “Table 2 displays the association between OBS and CVD risk, as well as the association between OBS and MMA”, but table 2 is entitled “Relationship of OBS and MMA with CVD risk among participants with hypertension”, and describe the associations between OBS and CVD risk, as well as the association between MMA and CVD. Please correct it.

Reply: Thanks very much for your suggestion. We have modified the content in the Result section: Table 2 displays the associations of OBS and MMA with CVD risk by the weighted multivariable logistic regression models.

9. Vitamin B12 was considered as a cofounder in the methodology; however, it was not included in the association analysis process. I recommend providing a proper explanation.

Reply: We sincerely appreciate your valuable comments. In this study, weighted univariable logistic regression analysis was used to screen the potential confounding factors that associated with CVD risk (Supplementary Table 1), and *P*<0.05 was considered significant. As shown in Supplementary Table 1, the impact of vitamin B_12_ on CVD did not reach statistical significance; therefore, it was not considered as a confounding factor in this study.

10. The reported effect size and CI, in line 191, are not correct. Please revise it.

Reply: Thanks very much for your suggestion. We have modified the content in the Result section: After further adjusting for MMA based on Model II ^(a)^, the association between OBS and CVD was significant [direct effect; Model II ^(b)^ OR=1.50, 95%CI: 1.15-1.96].

Discussion
11. Lines 218-219, the authors stated “To our knowledge, several epidemiological studies have explored the association between OBS and CVD risk”, yet only two articles were used to discuss the association between OBS and CVD. The same issue is observed with the association between MMA and CVD risk. As mentioned above these associations have been largely investigated, therefore the authors need to improve the discussion section. The discussion needs to be deepened, as it represents an important part of the manuscript, allowing both criticism and bringing more value.

Reply: We sincerely appreciate your valuable comments. According to your advice, we have modified the content in the discussion section: To our knowledge, several epidemiological studies have explored the association between OBS and CVD risk. Low OBS score may increase the risk of CVD (1, 2). OBS serves as an indicator of measuring body’s oxidant-antioxidant balance. Disruption of the pro-oxidant-antioxidant balance could result in OS and inflammation (3). It is widely recognized that OS and inflammation are closely related to the onset and development of CVD events (4). Furthermore, a low OBS score indicates an advantage of pro-oxidant over antioxidant exposure, resulting in elevated levels of OS. OS represents a cascade of adaptive responses triggered by an imbalance between ROS and the antioxidant system (5). ROS readily reacts with small molecules like low-density lipoprotein (LDL), leading to the formation of oxidized low-density lipoprotein (ox-LDL) (6). Driven by chemokines, such as stroma-derived factors and macrophage chemokines, ox-LDL circulates directly to the heart and blood vessels. Under the influence of adhesion factors like plasminogen activator inhibitors, these ox-LDL particles adhere to vascular walls, promoting arteriosclerosis and narrowing which ultimately the CVD development (7, 8). However, to the best of our knowledge, the majority of current studies have primarily focused on investigating the association between OBS and CVD risk within the general population. However, limited research has been conducted to explore whether OBS also exerts an impact on CVD risk among individuals with hypertension.

[1] Chen X, Wang C, Dong Z, Luo H, Ye C, Li L, et al. Interplay of Sleep Patterns and Oxidative Balance Score on Total Cardiovascular Disease Risk: Insights from the National Health and Nutrition Examination Survey 2005-2018. J Glob Health (2023) 13:04170. Epub 2023/12/12. doi: 10.7189/jogh.14.04170.

[2] Chen K, Li S, Xie Z, Liu Y, Li Y, Mai J, et al. Association between Oxidative Balance Score, Systemic Inflammatory Response Index, and Cardiovascular Disease Risk: A Cross-Sectional Analysis Based on Nhanes 2007-2018 Data. Front Nutr (2024) 11:1374992. Epub 2024/06/20. doi: 10.3389/fnut.2024.1374992.

[3] Qu H. The Association between Oxidative Balance Score and Periodontitis in Adults: A Population-Based Study. Front Nutr (2023) 10:1138488. Epub 2023/05/16. doi: 10.3389/fnut.2023.1138488.

[4] Pickering RJ. Oxidative Stress and Inflammation in Cardiovascular Diseases. Antioxidants (Basel) (2021) 10(2). Epub 2021/01/29. doi: 10.3390/antiox10020171.

[5] Pu Y, Cheng CK, Zhang H, Luo JY, Wang L, Tomlinson B, Huang Y. Molecular mechanisms and therapeutic perspectives of peroxisome proliferator-activated receptor α agonists in cardiovascular health and disease. Med Res Rev (2023) 43(6):2086-2114. Epub 2023/04/29. doi: 10.1002/med.21970.

[6] Gao S, Zhao D, Wang M, Zhao F, Han X, Qi Y, Liu J. Association Between Circulating Oxidized LDL and Atherosclerotic Cardiovascular Disease: A Meta-analysis of Observational Studies. Can J Cardiol (2017) 33(12):1624-1632. Epub 2017/07/29. doi: 10.1016/j.cjca.2017.07.015.

[7] Wang R, Gao K, Wang L, Gong X, Wu Y, Zheng L, et al. A Cross-Sectional Study Exploring the Relationship between Oxidative Balance Score and 10-Year Atherosclerotic Cardiovascular Disease Risk Based on the National Health and Nutrition Examination Survey (2011-2020). Diab Vasc Dis Res (2024) 21(2):14791641241244658. Epub 2024/04/10. doi: 10.1177/14791641241244658.

[8] Marchio P, Guerra-Ojeda S, Vila JM, Aldasoro M, Victor VM, Mauricio MD. Targeting Early Atherosclerosis: A Focus on Oxidative Stress and Inflammation. Oxid Med Cell Longev (2019) 2019:8563845. Epub 2019/07/30. doi: 10.1155/2019/8563845.

12. Please reform the sentences in lines 243-246 for more formality.

Reply: Thanks very much for your suggestion. We have modified the sentence in the discussion section: This finding also serves as a reminder of the significance of moderately reducing the MMA level to mitigate the onset of CVD, particularly in hypertensive populations with diets and lifestyles rich in pro-oxidants.

13. In line 250, the authors stated the effect of MMA mediating impact on the dyslipidemia group and not in the diabetes group, however, this result needs more discussion. I suggest that you provide plausible explanations for this part.

Reply: Thanks very much for your suggestion. According to your advice, we have added the content in the discussion section: For the hypertensive population along with dyslipidemia, MMA may partially mediate the association between OBS and CVD risk with a mediated proportion of 14.9%. This reason may be due to the mitochondrial redox dysfunction and mitochondrial energy metabolism abnormalities in population with dyslipidemia [1]. Moreover, our investigation also revealed that over 80% of the study population exhibited dyslipidemia.

[1] Oliveira HCF, Vercesi AE. Mitochondrial bioenergetics and redox dysfunctions in hypercholesterolemia and atherosclerosis. Mol Aspects Med. 2020; 71:100840.

14. Instead of only listing the limitations, I advise the authors to also list the strength of the present study.

Reply: Thanks very much for your suggestion. According to your advice, we have added the content in the discussion section: Our research had several advantages. Firstly, this was the first study to examine the association of between OBS, MMA, and CVD risk, and the mediating role played by MMA within participants with hypertension. Secondly, we utilized the nationally renowned NHANES database, which is widely recognized for its extensive sample size and representation of the entire nation. Thirdly, the association was found to be robust even after adjusting for various important confounders.

Conclusion
15. Please reform the conclusion section.
Additionally, please revise the overall manuscript for the language.

Reply: Thanks very much for your suggestion. According to your advice, we have added the content in the conclusion section: In summary, our study revealed the significant associations between lower OBS and an elevated risk of CVD, as well as an increased level of MMA among participants with hypertension. The higher MMA was found to be associated with an increased possibility of CVD. Of particular significance, MMA plays a critical mediating role in the pathway between OBS and CVD risk.

Additionally, we have checked and revised the overall manuscript for the language.

a. Is the quality of the figures and tables satisfactory?
- Yes
Reply: Thanks very much for your review.

b. Does the reference list cover the relevant literature adequately and in an unbiased manner?
- Yes
Reply: Thanks very much for your review.

c. Are the statistical methods valid and correctly applied? (e.g. sample size, choice of test)
- Yes
Reply: Thanks very much for your review.

d. Is a statistician required to evaluate this study?
- Yes

Reply: Thanks very much for your review. Our study has been evaluated by a statistician. Please see the revised manuscript.

e. Are the methods sufficiently documented to allow replication studies?
- Yes

Reply: Thanks very much for your review.
